# Estudo Prospectivo do Perfil Lipídico em Pacientes Incidentes em Diálise Peritoneal

**DOI:** 10.36660/abc.20260013

**Published:** 2026-07-30

**Authors:** Luiza K. R. P. Araújo, Dayana B. Dias, Monique F. M. Santana, Benedito J. Pereira, Hugo Abensur, Rosa Moyses, Marisa Passarelli, Rosilene M. Elias

**Affiliations:** 1 Hospital das Clinicas da Faculdade de Medicina da Universidade de São Paulo São Paulo SP Brasil Nefrologia, Hospital das Clinicas da Faculdade de Medicina da Universidade de São Paulo (HCFMUSP), São Paulo, SP – Brasil; 2 Universidade Estadual Paulista Botucatu SP Brasil Universidade Estadual Paulista, Botucatu, SP – Brasil; 3 Hospital das Clínicas da Faculdade de Medicina da Universidade de São Paulo Laboratório de Lípides São Paulo SP Brasil Laboratório de Lípides (LIM10), Hospital das Clínicas da Faculdade de Medicina da Universidade de São Paulo (HCFMUSP), São Paulo, SP – Brasil; 4 Universidade Nove de Julho Programa de Pós-Graduação em Medicina São Paulo SP Brasil Programa de Pós-Graduação em Medicina, Universidade Nove de Julho, São Paulo, SP – Brasil

**Keywords:** Falência Renal Crônica, Dislipidemia, Colesterol

## Introdução

A dislipidemia é uma complicação metabólica comum em pacientes com doença renal crônica (DRC).^1^ Embora os mecanismos subjacentes não sejam totalmente compreendidos, acredita-se que o estresse oxidativo, a inflamação e a resistência à insulina contribuam para a aterosclerose acelerada.

A diálise peritoneal (DP) é uma terapia domiciliar para pacientes com DRC avançada. Nessa modalidade, a glicose é o principal agente osmótico na solução de diálise, levando a efeitos metabólicos sistêmicos.^2,3^ Pacientes submetidos à DP tendem a apresentar um perfil lipídico mais aterogênico do que aqueles que recebem tratamento conservador ou hemodiálise.^4^ Esse perfil aterogênico é gerado tanto pela perda de proteínas no solução de diálise quanto pelo uso de soluções de diálise com alto teor de glicose, que estimulam citocinas que promovem inflamação crônica.^5^

Normalmente, pacientes em DP apresentam níveis elevados de triglicerídeos, lipoproteína de densidade muito baixa (VLDL), lipoproteína de baixa densidade (LDL), colesterol total e lipoproteína(a) [Lp(a)], enquanto os níveis de lipoproteína de alta densidade (HDL) tendem a ser baixos.^6^

Embora a dislipidemia em pacientes em DP tenha sido descrita na literatura, os estudos prospectivos são limitados e envolvem principalmente pacientes prevalentes nessa modalidade. Consequentemente, o verdadeiro impacto da DP nos perfis lipídicos permanece incerto. Além disso, o uso disseminado de estatinas nessa população levanta questionamentos sobre se a DP ainda influencia negativamente o metabolismo lipídico na prática clínica atual. Esclarecer essa questão é crucial, pois pode orientar estratégias de tratamento, apoiar o planejamento de ensaios clínicos e, potencialmente, aumentar a adoção da DP como modalidade de diálise. O objetivo do presente estudo foi descrever as alterações longitudinais nos perfis lipídicos e identificar os fatores associados em uma coorte de pacientes incidentes em DP durante os primeiros seis meses de terapia.

## Métodos

Este foi um estudo de coorte prospectivo multicêntrico de pacientes incidentes em DP, realizado entre abril de 2019 e junho de 2024. Incluímos pacientes adultos que iniciaram terapia de DP durante o período do estudo e os acompanhamos por 6 meses. Os pacientes foram recrutados em dois centros acadêmicos (Universidade de São Paulo, São Paulo, e Universidade Estadual Paulista, Botucatu). Excluímos pacientes que migraram da hemodiálise para a DP e aqueles com diabetes mellitus. Para reduzir o viés de aferição, garantimos que as dosagens de colesterol fossem realizadas utilizando os mesmos métodos em ambos os centros. A variável de interesse foi o colesterol total, avaliado no início do estudo e após 6 meses de terapia.

O uso de estatinas foi tratado como uma variável de confusão. Dados demográficos, clínicos e laboratoriais foram avaliados durante as consultas médicas. De acordo com o teste de Shapiro-Wilk, todas as variáveis, exceto idade e ureia, apresentaram distribuição não normal e, portanto, foram apresentadas como mediana (percentis 25-75). As comparações entre o período basal e 6 meses após o início da DP foram realizadas utilizando o teste de Wilcoxon para variáveis contínuas e o teste de McNemar para variáveis categóricas, conforme apropriado.

O nível de significância adotado foi de 5%. As análises estatísticas foram realizadas utilizando o SPSS versão 29.0 (SPSS Inc., Chicago, IL, EUA) e o GraphPad Prism^®^ versão 10 do software (GraphPad Software, Inc., CA, EUA).

## Resultados

As características dos pacientes estão descritas na Tabela 1. Após 6 meses de DP, os pacientes apresentaram um aumento de peso corporal, sem alterações significativas em colesterol total, colesterol LDL ou colesterol HDL. Os níveis séricos de triglicerídeos diminuíram significativamente. Não foi aplicado nenhum ajuste formal para multiplicidade nas análises secundárias, e os resultados devem ser interpretados com cautela.

**Tabela 1:** Valores basais e de 6 meses para peso e perfil lipídico

		Linha de base N=47	6 meses N=47	p
**Idade (anos)**		50,5 ± 19,7	-	
**Sexo masculino (%)**		44,7	-	
**Peso (kg)**		64,1 (53,6-74,9)	66,7 (58,4-75,2)	0,001
**Etiologia da DRC, %**				
	Glomerulonefrite	42,6	-	
	Nefrosclerose	42,6	-	
	Outro/desconhecido	14,9	-	
**Infarto do miocárdio prévio, %**		10.6	-	
**Insuficiência cardíaca congestiva, %**		8,5	-	
**Acidente vascular cerebral prévio, %**		2.1	-	
**Tabagismo, %**		8,5	-	
**Medicação em uso (%)**				1
	Estatina	74,5	74,5	
	Fibrato	2.1	2.1	
	Hidroclorotiazida	21.3	21.3	
	Furosemida	78,7	78,7	
**Ureia, mg/dL**		123±39	-	
**Creatinina, mg/dL**		5,5 (4,5-7,3)	-	
**Colesterol total (mg/dl)**		178 (140-227)	160 (139-220)	0,449
	LDL-c (mg/dl)	97 (77-129)	88,5 (76-139)	0,698
	HDL-c (mg/dl)	43 (34-58)	42 (38-54)	0,957
**Triglicerídeos (mg/dl)**		169 (101-218)	127 (101-162)	0,005

Os dados são expressos como mediana (percentil 25 - percentil 75). CT: colesterol total; LDL-c: colesterol de lipoproteína de baixa densidade; HDL-c: colesterol de lipoproteína de alta densidade.

O comportamento de peso corporal da linha de base até os 6 meses é mostrado na Figura 1A. A variação mediana em colesterol total (Figura 1B) foi de −7 mg/dl, com um aumento observado em 22 pacientes (46,8%). Os pacientes que apresentaram aumento no colesterol total tinham prescrições de estatinas semelhantes às daqueles que apresentaram diminuição ou nenhuma alteração significativa (63,6% vs. 84,0%, p = 0,110), mas eram mais propensos a receber hidroclorotiazida (36,8% vs. 8,0%, p = 0,018). Uma análise de regressão linear sobre os fatores associados à mudança em colesterol total (variável dependente) mostrou que o uso de estatinas (coeficiente beta padronizado −0,336, IC 95% -69,90 a -8,33, correlação parcial 0,368, p=0,014) e o uso de hidroclorotiazida (coeficiente beta padronizado 0,285, IC 95% 0,94 a 67,86, correlação parcial 0,305, p=0,044), mas não o ganho de peso (coeficiente beta padronizado 0,193, IC 95% -1,39 a 7,79, correlação parcial 0,212, p=0,167), estiveram associados à variação do colesterol total em 6 meses. Juntas, essas variáveis explicaram 23,4% da variação do colesterol (R² total do modelo = 0,534, p=0,003). A inclusão de idade e sexo no modelo não alterou os resultados.

**Figura 1:**
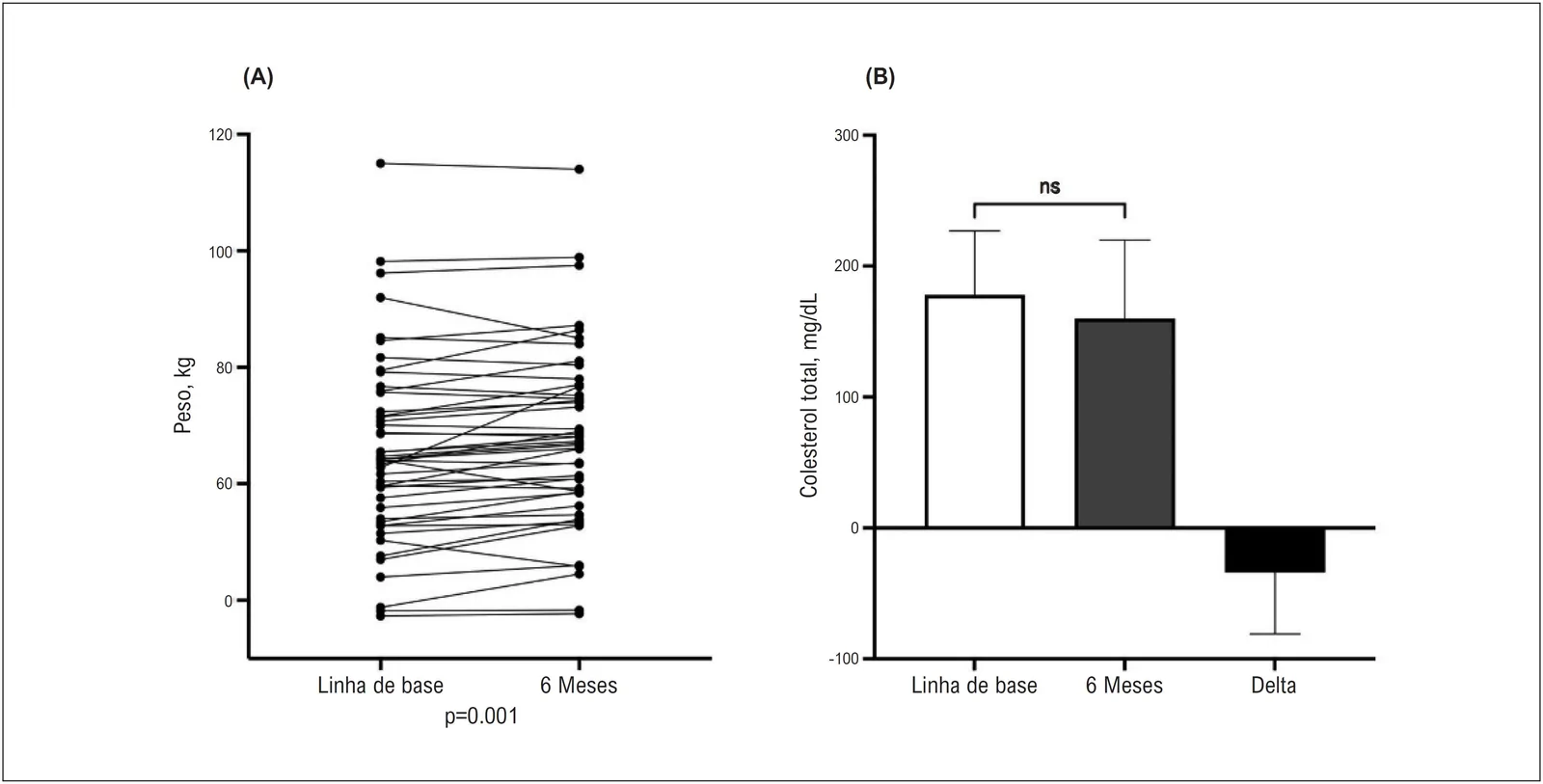
Comportamento do peso corporal (A) e variação mediana do colesterol total (B).

## Discussão

Este estudo sugere que os níveis de colesterol total não aumentam em pacientes incidentes em DP durante os primeiros 6 meses de terapia, apesar do aumento de peso corporal. Notavelmente, as prescrições de estatinas permaneceram inalteradas durante todo o período do estudo, o que pode ter contribuído para esse achado.

A dislipidemia é um fator de risco cardiovascular bem estabelecido tanto na população geral^7,8^ quanto em pacientes com DRC.^9^ Em pacientes em DP, a dislipidemia tende a ser mais aterogênica, principalmente devido ao aumento dos níveis de colesterol total.^10^ Poder-se-ia esperar, devido ao conteúdo de glicose do dialisato e ao ganho de peso que tipicamente ocorre durante a terapia de DP, que os níveis de colesterol aumentassem nessa população. De fato, um estudo anterior demonstrou uma melhora no perfil lipídico quando um regime de DP com redução da exposição à glicose foi utilizado.^11^ Em contraste, outro estudo apontou o ganho de peso, em vez de produtos à base de glicose, como o principal contribuinte para a piora dos perfis lipídicos em pacientes em DP.^12^ Estudos retrospectivos que avaliaram as tendências do colesterol em pacientes em DP mostraram estabilidade ao longo do tempo.^13,14^ Embora diversos estudos tenham investigado a associação entre os níveis de colesterol e a mortalidade em pacientes prevalentes^15^ e incidentes em DP,^16,17^ os dados sobre alterações longitudinais no perfil lipídico permanecem limitados. Um estudo prospectivo que incluiu 39 pacientes incidentes em DP não relatou nenhuma alteração significativa nos níveis de colesterol durante os primeiros seis meses de terapia, o que está de acordo com nossos achados.^18^ Resultados semelhantes foram observados em uma subanálise do estudo balANZ.^6^ No entanto, a ausência de alteração nos níveis de colesterol não implica necessariamente uma melhora no risco aterosclerótico, visto que outros biomarcadores ainda podem ser afetados negativamente.

No presente estudo, metade dos pacientes apresentou aumento do colesterol total após seis meses de DP, o que foi associado ao uso de hidroclorotiazida. Essa descoberta não contradiz o desfecho primário, mas representa uma análise exploratória adicional. Mais estudos são necessários para confirmar essa associação em pacientes em DP, semelhante aos achados relatados em pacientes com hipertensão.^19,20^ A redução observada nos níveis de triglicerídeos, apesar do ganho de peso, pode ser explicada por mudanças na composição corporal (massa muscular versus condição hipervolêmica), modificações dietéticas após o início da diálise ou melhor controle metabólico devido à redução da inflamação e à melhora da resistência à insulina.

Este estudo apresenta algumas limitações, incluindo um tamanho amostral pequeno, um curto período de acompanhamento e a ausência de um grupo controle. No entanto, dada a escassez de dados prospectivos em pacientes submetidos à DP, acreditamos que este estudo adiciona evidências significativas à literatura existente. Se a estabilidade dos níveis de colesterol total em pacientes em DP se traduz em redução da mortalidade cardiovascular justifica uma investigação mais aprofundada.

## Data Availability

Os conjuntos de dados gerados e/ou analisados durante o presente estudo não estão disponíveis publicamente devido a restrições éticas e de confidencialidade dos participantes. Entretanto, poderão ser disponibilizados pelo autor correspondente mediante solicitação razoável e após aprovação do Comitê de Ética em Pesquisa.
