# Supplementary material for: Estudo Prospectivo do Perfil Lipídico em Pacientes Incidentes em Diálise Peritoneal
Source: Arq Bras Cardiol. 2026 Jul 30;123(7):e20260013. [Article in Portuguese] doi: 10.36660/abc.20260013 (PMC13668456; doi:10.36660/abc.20260013)
Supplement: Translation

# Translation: A Prospective Study of Lipid Profile in Incident Patients on Peritoneal Dialysis

**DOI:** 10.36660/abc.20260013i

**Authors:** Luiza K. R. P. Araújo, Dayana B. Dias, Monique F. M. Santana, Benedito J. Pereira, Hugo Abensur, Rosa Moyses, Marisa Passarelli, Rosilene M. Elias

**Affiliations:** 1 São Paulo SP Brasil Nefrologia, Hospital das Clinicas da Faculdade de Medicina da Universidade de São Paulo (HCFMUSP), São Paulo, SP – Brasil; 2 Botucatu SP Brasil Universidade Estadual Paulista, Botucatu, SP – Brasil; 3 São Paulo SP Brasil Laboratório de Lípides (LIM10), Hospital das Clínicas da Faculdade de Medicina da Universidade de São Paulo (HCFMUSP), São Paulo, SP – Brasil; 4 São Paulo SP Brasil Programa de Pós-Graduação em Medicina, Universidade Nove de Julho, São Paulo, SP – Brasil

**Keywords:** Chronic Kidney Failure, Dyslipidemias, Cholesterol

## Introduction

Dyslipidemia is a common metabolic complication in patients with chronic kidney disease (CKD).^1^ Although the underlying mechanisms are not fully understood, oxidative stress, inflammation, and insulin resistance are thought to contribute to accelerated atherosclerosis.

Peritoneal dialysis (PD) is a home-based therapy for patients with advanced CKD. In this modality, glucose is the predominant osmotic agent in the dialysate, leading to systemic metabolic effects.^2,3^ Patients undergoing PD tend to have a more atherogenic lipid profile than those receiving conservative treatment or hemodialysis.^4^ This atherogenic profile is generated both by protein loss in the dialysate and by the use of high-glucose dialysis solutions that stimulate cytokines promoting chronic inflammation.^5^

Typically, patients on PD have elevated levels of triglycerides, very low-density lipoprotein (VLDL), low-density lipoprotein (LDL), total cholesterol, and lipoprotein(a) [Lp(a)], whereas high-density lipoprotein (HDL) levels tend to be low.^6^

Although dyslipidemia in patients on PD has been described in the literature, prospective studies are limited and mostly involve prevalent patients on this modality. As a result, the true impact of PD on lipid profiles remains unclear. In addition, the widespread use of statins in this population raises questions about whether PD still negatively influences lipid metabolism in contemporary practice. Clarifying this issue is crucial, as it may inform treatment strategies, support the design of clinical trials, and potentially increase the adoption of PD as a dialysis modality. The objective of the present study was to describe longitudinal changes in lipid profiles and identify associated factors in a cohort of incident PD patients during the first six months of therapy.

## Methods

This was a prospective multicenter cohort study of incident patients on PD conducted between April 2019 and June 2024. We included adult patients who initiated PD therapy during the study period and followed them for 6 months. Patients were recruited from two academic centers (Universidade de São Paulo, São Paulo, and Universidade Estadual Paulista, Botucatu). We excluded patients who switched from hemodialysis to PD and those with diabetes mellitus. To reduce measurement bias, we ensured that cholesterol measurements were performed using the same methods in both centers. The variable of interest was total cholesterol, evaluated at baseline and after 6 months of therapy.

Statin use was treated as a confounding variable. Demographic, clinical, and laboratory data were assessed during physician consultations. According to the Shapiro-Wilk test, all variables, except age and urea, showed a non-normal distribution and were therefore presented as median (25^th^-75^th^ percentile). Comparisons between baseline and 6 months after PD initiation were performed using the Wilcoxon signed-rank test for continuous variables and the McNemar test for categorical variables, as appropriate.

The significance level adopted was 5%. Statistical analyses were performed using SPSS version 29.0 (SPSS Inc., Chicago, IL, USA) and GraphPad Prism^®^ software version 10 (GraphPad Software, Inc., CA, USA).

## Results

Characteristics of patients are described in Table 1. After 6 months on PD, patients experienced an increase in body weight, with no significant changes in total cholesterol, LDL-cholesterol, or HDL-cholesterol. Serum triglyceride levels significantly decreased. No formal multiplicity adjustment was applied to secondary analyses, and the findings should be interpreted cautiously.

**Table 1:** Baseline and 6-month values for weight and lipid profile

		Baseline N=47	6-month N=47	p
**Age (years)**		50.5 ± 19.7	-	
**Male sex (%)**		44.7	-	
**Weight (kg)**		64.1 (53.6-74.9)	66.7 (58.4-75.2)	0.001
**Etiology of the CKD, %**				
	Glomerulonephritis	42.6	-	
	Nephrosclerosis	42.6	-	
	Other/unknown	14.9	-	
**Previous myocardial infarction, %**		10.6	-	
**Congestive heart failure, %**		8.5	-	
**Previous stroke, %**		2.1	-	
**Smoking, %**		8.5	-	
**Medication in use (%)**				1
	Statin	74.5	74.5	
	Fibrate	2.1	2.1	
	Hydrochlorothiazide	21.3	21.3	
	Furosemide	78.7	78.7	
**Urea, mg/dL**		123 ± 39	-	
**Creatinine, mg/dL**		5.5 (4.5-7.3)	-	
**Total cholesterol (mg/dl)**		178 (140-227)	160 (139-220)	0.449
	LDL-c (mg/dl)	97 (77-129)	88.5 (76-139)	0.698
	HDL-c (mg/dl)	43 (34-58)	42 (38-54)	0.957
**Triglycerides (mg/dl)**		169 (101-218)	127 (101-162)	0.005

Data are expressed as median (25th percentile-75th percentile). TC: total cholesterol; LDL-c: low-density lipoprotein cholesterol; HDL-c: high-density lipoprotein cholesterol.

The behavior of body weight from baseline to 6 months is shown in Figure 1A. The median change in total cholesterol (Figure 1B) was −7 mg/dl, with an increase observed in 22 patients (46.8%). Patients who experienced an increase in total cholesterol had similar statin prescriptions compared with those who had a decrease or no significant change (63.6% vs. 84.0%, p = 0.110), but were more likely to be receiving hydrochlorothiazide (36.8% vs. 8.0%, p = 0.018). A linear regression analysis on the factors associated with change in total cholesterol (dependent variable) showed that statin use (std beta coefficient −0.336, 95% CI -69.90- to -8.33, partial correlation 0.368, p=0.014) and hydrochlorotiazide use (std beta coefficient 0.285, 95% CI 0.94 to 67.86, partial correlation 0.305, p=0.044), but not weight gain (std beta coefficient 0.193, 95% CI -1.39 to 7.79, partial correlation 0.212, p=0.167) were associated with change in total cholesterol in 6 months. Together, these variables explained 23.4% of the change in cholesterol (total model R=0.534, p=0.003). Inclusion of age and sex in the model did not change the results.

**Figure 1:** Body weight behavior (A) and median change in total cholesterol (B).

## Discussion

This study suggests that total cholesterol levels do not increase in incident patients undergoing PD during the first 6 months of therapy, despite an increase in body weight. Notably, statin prescriptions remained unchanged throughout the study period, which may have contributed to this finding.

Dyslipidemia is a well-established cardiovascular risk factor in both the general population^7,8^ and patients with CKD.^9^ In patients on PD, dyslipidemia tends to be more atherogenic, mainly due to increased total cholesterol levels.^10^ One might expect, due to the glucose content of the dialysate and the weight gain that typically occurs during PD therapy, that cholesterol levels would increase in this population. Indeed, a previous study demonstrated an improvement in lipid profile when a glucose-sparing PD regimen was used.^11^ In contrast, another study implicated weight gain, rather than glucose-based products, as the main contributor to worsening lipid profiles in patients on PD.^12^ Retrospective studies evaluating cholesterol trends in patients on PD have shown stability over time.^13,14^ Although several studies have investigated the association between cholesterol levels and mortality in both prevalent^15^ and incident patients on PD,^16,17^ data on longitudinal changes in lipid profile remain limited. One prospective study that included 39 incident patients on PD reported no significant change in cholesterol levels during the first six months of therapy, consistent with our findings.^18^ Similar results were observed in a subanalysis of the balANZ trial.^6^ However, the absence of change in cholesterol levels does not necessarily imply an improvement in atherosclerotic risk, as other biomarkers may still be adversely affected.

In the current study, half of the patients showed an increase in total cholesterol after six months of PD, which was associated with the use of hydrochlorothiazide. This finding does not contradict the primary outcome, but rather represents an additional exploratory analysis. Further studies are needed to confirm this association in patients on PD, similar to findings reported in patients with hypertension.^19,20^ The observed reduction in triglyceride levels despite weight gain may be explained by changes in body composition (muscle mass vs. hypervolemic condition), dietary modifications after dialysis initiation, or improved metabolic control due to reduced inflammation and improved insulin resistance.

This study has some limitations, including a small sample size, a short follow-up period, and the absence of a control group. Nevertheless, given the scarcity of prospective data in patients undergoing PD, we believe this study adds meaningful evidence to the existing literature. Whether the stability of total cholesterol levels in patients on PD translates into reduced cardiovascular mortality warrants further investigation.

## Data Availability

The datasets generated and/or analyzed during the current study are not publicly available due to ethical and patient confidentiality restrictions but are available from the corresponding author on reasonable request, subject to approval by the Institutional Review Board.
